# Natural Spawning, Early Development, and First Successful Hatchery Production of the Vermiculated Angelfish (*Chaetodontoplus mesoleucus*), Exploring the Influence of Temperature and Salinity

**DOI:** 10.3390/ani15111657

**Published:** 2025-06-04

**Authors:** Yu-Hsuan Sun, Yu-Ru Lin, Hung-Yen Hsieh, Pei-Jie Meng

**Affiliations:** 1 Graduate Institute of Marine Biology, College of Environment Studies and Oceanography, National Dong Hwa University, Hualien 974301, Taiwan; angel750508@gmail.com; 2 Department of Aquaculture, College of Life Science, National Taiwan Ocean University, Keelung 202301, Taiwan; linyuru09@gmail.com; 3 Taiwan Ocean Research Institute (TORI), National Institutes of Applied Research, Kaohsiung 85243, Taiwan; 4 Department of Oceanography, National Sun Yat-sen University, Kaohsiung 80424, Taiwan; 5 Research Center for Critical Issues, Academia Sinica, Taipei 11529, Taiwan

**Keywords:** *Chaetodontoplus mesoleucus* (Bloch, 1787), larviculture, embryonic and larval culture, juvenile, temperature and salinity

## Abstract

The marine ornamental species trade relies heavily on wild-caught specimens, including the Vermiculated angelfish (*Chaetodontoplus mesoleucus*). Captive breeding of this species faces challenges with limited detailed knowledge available beyond 2 days post-hatch (dph) regarding the influence of environmental factors. This study provides a detailed characterization of *C. mesoleucus* from early development to 381 dph. Under controlled laboratory conditions, the effect of temperature (22–37 °C) on hatching rate, deformity rate, hatching period duration, time to 50% hatch, and survival rate is investigated. Additionally, the influence of different salinities (0–38 psu) on hatching rates and larval deformity rates was also examined. The optimal incubation temperatures for high hatching rate and minimal larval deformities are found to be within 25–28 °C. A lower salinity threshold of 10 psu was established for successful hatching, and the optimal salinity range for minimizing larval deformities was 33–36 psu. These findings provide crucial baseline data and practical recommendations for optimizing hatchery protocols for *C. mesoleucus*, contributing to enhanced larval survival and the potential for sustainable aquaculture production, thereby reducing the pressure on wild populations.

## Simple Summary

The Vermiculated angelfish (*Chaetodontoplus mesoleucus*) is a popular species in the global marine aquarium trade. While it is commonly sourced from the wild, the methods of collection, such as the use of cyanide or destructive harvesting techniques, can negatively impact coral reef ecosystems. Breeding these fish in captivity is difficult, especially in the early stages of their development. This study successfully accomplished the first artificial breeding and rearing of the Vermiculated angelfish. This study looked closely at how these fish develop from eggs to young larvae and how different water temperatures and salt levels affect them. We found out more about their early growth than was previously known. We also suggest the best temperature range for their eggs to hatch into healthy larvae and the right salt levels needed for successful development. This information is important for people trying to breed these fish in tanks. By understanding the best conditions for their early life, we can improve breeding methods, reduce the need to catch them in the wild, and help protect their natural environment on coral reefs, making the ornamental fish trade more sustainable.

## 1. Introduction

The Vermiculated angelfish, *Chaetodontoplus mesoleucus* (Bloch, 1787), a member of the family Pomacanthidae, is a popular marine ornamental fish. It is widely distributed in Indo-West Pacific coral reefs (Ryukyu Islands to Papua New Guinea) and feeds primarily on sessile invertebrates [1]. Its striking coloration drives high market demand, positioning *C. mesoleucus* as a frequently traded species in the marine ornamental fish industry. The industry’s heavy reliance on wild capture, accounting for over 98% of specimens including *C. mesoleucus*, raises significant concerns regarding sustainability and potential conservation impacts. In the 2024 CITES technical workshop on marine ornamental fishes, *C. mesoleucus* was categorized as a “moderately susceptible” species to collection pressure, indicating that while it currently tolerates fishing’s impact to some extent, it has experienced measurable impacts and warrants close monitoring [2]. Unlike freshwater ornamental species, predominantly produced through aquaculture, only about 10% of marine ornamental species have been successfully bred in captivity [3]. With increasing demand [4], developing reliable aquaculture techniques is crucial for alleviating the pressure on wild populations and ensuring the long-term sustainability of the industry. Establishing effective captive breeding protocols offers a viable alternative to wild collection and plays a vital role in mitigating the ecological consequences of overexploitation [5].

The artificial propagation of *C. mesoleucus* presents considerable challenges, particularly for optimizing environmental conditions during the critical embryonic and larval development stages. Despite advancements in marine ornamental aquaculture since the 1970s, commercial production remains nascent [6], with fewer than 30 of the hundreds of successfully bred species reaching stable commercial production [7]. Larval rearing is a persistent bottleneck in marine ornamental aquaculture, even after successful artificial spawning [8,9]. Previous work by Fujita and Mito [10] achieved induced natural spawning of *C. mesoleucus* in captivity, reporting egg diameters of 0.95–1.05 mm. However, the newly hatched larvae survived only until 5 dph, indicating significant challenges in early-stage rearing, likely due to limitations in both nutrition and environmental parameters. Early-stage larvae are known to be extremely fragile, exhibiting high sensitivity to water quality fluctuations [9,11] and requiring a nutritionally optimized diet [12,13]. Key abiotic factors such as temperature, salinity, dissolved oxygen, photoperiod, and pH must be meticulously controlled to mimic natural habitats, as these parameters critically influence larval survival and development [14,15]. However, detailed knowledge regarding the specific early developmental stages and the precise influence of key environmental factors like temperature and salinity on the embryonic and larval development of *C. mesoleucus* remains limited, particularly beyond the initial few days post-hatching [10].

Among the environmental factors, temperature is recognized as particularly influential, directly impacting developmental rates, embryo size, and overall survival [16,17]. Marine fish embryos and larvae are generally more sensitive to environmental fluctuations than adult fish [18], and suboptimal temperatures can delay development or induce malformations, with optimal hatching success typically observed within a narrow thermal range. Similarly, salinity stability is critical for embryo viability, with *C. mesoleucus* likely requiring near-full-strength seawater (32–34 ppt) for successful hatching and subsequent larval development. Dissolved oxygen levels must be maintained near saturation to avoid dramatically reduced hatching rates. Photoperiod also influences larval physiology, with inappropriate light exposure potentially increasing mortality and abnormalities, while an appropriate light–dark cycle can improve survival and development. Furthermore, maintaining pH within a stable seawater range (~7.9–8.4) is essential to prevent poor hatching success and deformities. Therefore, a comprehensive investigation into the early developmental trajectory of *C. mesoleucus* and the specific effects of temperature and salinity on its embryonic and larval stages is crucial for establishing effective aquaculture protocols and overcoming the current limitations in captive breeding.

This study aimed to address these knowledge gaps by providing a detailed characterization of the early developmental stages of *C. mesoleucus* beyond the previously documented 68 houses post-hatch (hph) [19]. Furthermore, we investigated the effects of a range of incubation temperatures and salinities on hatching success, larval deformity rates, and hatching dynamics. The findings of this research will contribute valuable insights into the fundamental biology of *C. mesoleucus* early life stages and provide practical information for the development of optimized aquaculture techniques for this important ornamental species.

## 2. Materials and Methods

### 2.1. Broodstock Rearing and Spawning

The broodstock comprised captive-bred progeny of wild *C. mesoleucus*—originating from multiple wild broodstock pairings—and had been maintained in a recirculating aquaculture system for approximately four years. Larger individuals were selected for pairing, forming a breeding group composed of one male (9–10 cm total length (TL)) and three females (7–8 cm TL). The broodstock was maintained in a 5-ton RAS under controlled indoor conditions. The environmental parameters were carefully regulated, including a photoperiod of 12 h L:12 h D, with salinity maintained at 32–34 psu, dissolved oxygen saturation exceeding 85%, a pH ranging from 7.9 to 8.3, and water temperature maintained between 24 and 29 °C; these items were measured daily with a multiparameter (YSI ProQUATRO, Yellow Springs, OH, USA). NH_4_-N and NO_2_-N concentrations were kept below 0.25 ppm and measured twice a week with a portable spectrophotometer (Hach DR900, Loveland, CO, USA). The feeding regimen consisted of one daily ration of frozen food (Antarctic krill, squid, oysters, and pacific saury meat), and two daily feedings of artificial feed to ensure adequate nutrition and reproductive health.

### 2.2. Egg Data Collection

Fertilized eggs were collected daily after 17:00 using an 80-mesh plankton net positioned at the outlet of the recirculating tank. Egg retrieval was completed before 20:00, and sinking eggs were removed. Floating eggs were examined under a dissecting microscope to assess normal embryonic development prior to use in the experiments.

### 2.3. Observations and Measurements of Fertilized Eggs, Larvae, and Juveniles

Fertilized eggs were placed in 2 L beakers, with the water temperature maintained at 27 ± 0.5 °C, consistent with the spawning tank conditions. Embryonic development was observed under an optical microscope (LEICA DM2000 LED, Wetzlar, Germany), and images were captured using a digital imaging system (LEICA MC190 HD, Wetzlar, Germany). Morphological characteristics, including egg shape, diameter, oil globule position, and yolk sac color and size, were analyzed following the methods of Lue et al. [20]. For daily larval sampling, 5–10 larvae were anesthetized with 2 ppm MS-222 (Sigma, St. Louis, MO, USA) for morphological observation and measurement under a dissecting microscope (LEICA S9D, Wetzlar, Germany). Measurements were conducted following the protocols described by Williams [21] and Lue et al. [22], including TL, standard length (SL), yolk-sac length (YL), oil globule diameter (OD), and gape height (GH). Gape height (GH) was calculated using the following equation:

$$\mathrm{GH}=\sqrt{\mathrm{UJL}+\mathrm{LJL}}$$

where UJL represents upper jaw length, and LJL represents lower jaw length. Yolk sac and oil globule consumption were estimated using the following volume formula:

$$V=\frac{4}{3}\pi r_{1}r_{2}r_{3}$$

where V represents volume (mm^3^), and r_1_, r_2_, and r_3_ correspond to the half-length, width, and height of the yolk sac and oil globule, respectively [21].

### 2.4. Effects of Temperature on Hatch Rate, Time to 50% Hatch, Hatching Period Duration, and Deformity Rate

The experiment was designed to evaluate the effects of temperature on hatching rate and early larval development. Six temperature treatments, set at 22 °C, 25 °C, 28 °C, 31 °C, 34 °C, and 37 °C, were conducted using 2 L beakers, with three replicates per treatment. Each beaker was stocked with 50 fertilized eggs. Temperature was precisely regulated using a thermostatic circulating water bath (YIH DER, BL-720D-20, Taipei, Taiwan), while salinity was maintained at 33 ± 0.5 psu, and a 12 h L:12h D photoperiod was applied throughout the experiment. Fertilized eggs were collected before reaching the blastula stage and incubated in each treatment group. The temperature was gradually adjusted from the original spawning tank condition (27 ± 0.5 °C) at a rate of 1 °C per 20 min until the target temperature was reached. The hatching rate was calculated. The 50% hatching time required for 50% of the larvae to hatch in each treatment was recorded. The hatching period duration was recorded from the first larval hatch to the last one. After hatching, the hatch rate, deformity rate, time to 50% hatch, hatching period duration, and survival rate were recorded following the methodologies of Hart [23] and Gracia-Lopez [24]. The hatch rate, deformity rate, and survival rate were calculated using the following equations:

$$Hatch\;rate\;(\%)=(\frac{Number\;of\;hatching\;larvae}{Total\;initial\;number\;of\;fertilized\;egg})⨯100$$

$$Deformity\;rate\;(\%)=(\frac{Number\;of\;deformed\;larvae}{Total\;hatched\;larvae})⨯100$$

$$Survival\;Rate\;(\%)=(\frac{Number\;of\;surviving\;larvae\;at\;3\;dph}{Number\;of\;hatched\;larvae\;at\;0\;dph})⨯100$$

### 2.5. Effects of Salinity on Hatch Rate and Deformity Rate

The experiment consisted of 5 salinity treatments: 24 psu, 27 psu, 30 psu, 33 psu, and 36 psu for the deformity rate experiment and 10 salinity treatments: 0 psu, 6 psu, 10 psu, 14 psu, 18 psu, 22 psu, 26 psu, 30 psu, 34 psu and 38 psu for the hatch rate experiment, both conducted in 2 L beakers with three replicates per treatment. Temperature was maintained at 27 ± 0.5 °C with a 12 h L:12 h D photoperiod. Fertilized eggs were collected from the spawning tank and directly placed into the respective salinity treatments (50 eggs per treatment). Hatch rate and deformity rate were recorded 22–24 h post-hatch using the same calculation methods as in the temperature experiment. The experiment followed the methodology of Hart [23] and Gracia-Lopez [24].

### 2.6. Statistical Analyses

The analyses data were expressed as means ± standard deviations (SDs). The data were tested for significance by one-way analyses of variance (ANOVA) followed and, where appropriate, by Tukey’s multiple comparison test, using GraphPad Prism 10.4.0. Detailed descriptive statistics—including means, maxima, minima, variances, and standard errors—of all tests performed are provided in Supplementary Tables S1–S7.

## 3. Results

### 3.1. Embryonic Development

**Figure 1:**
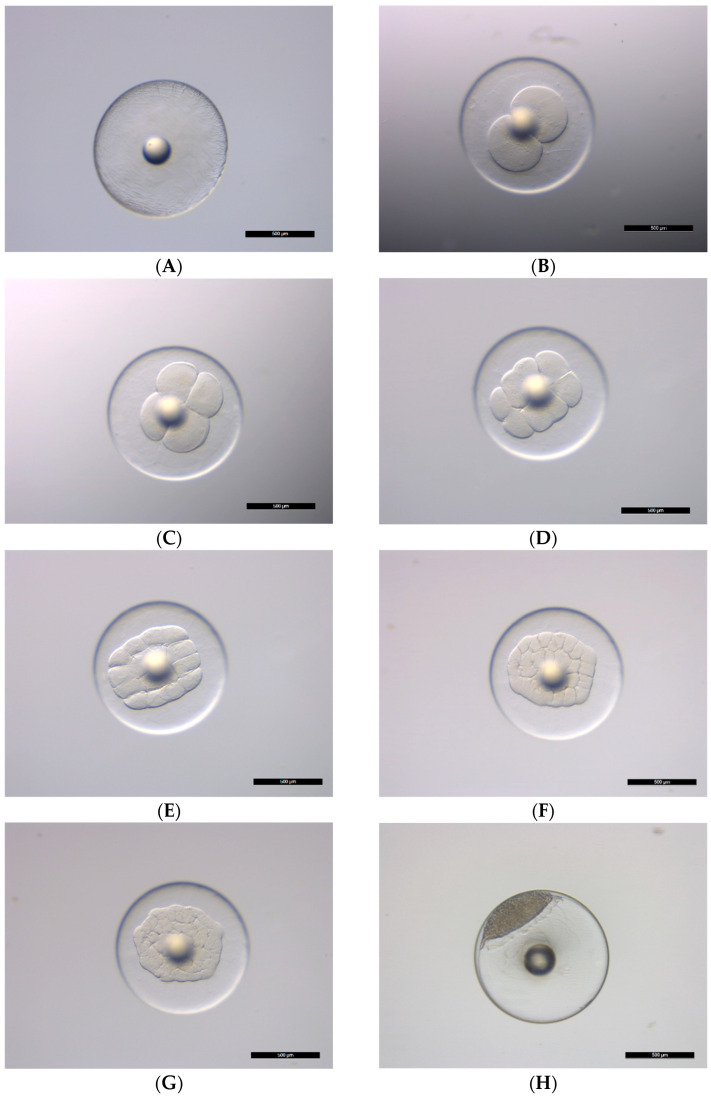

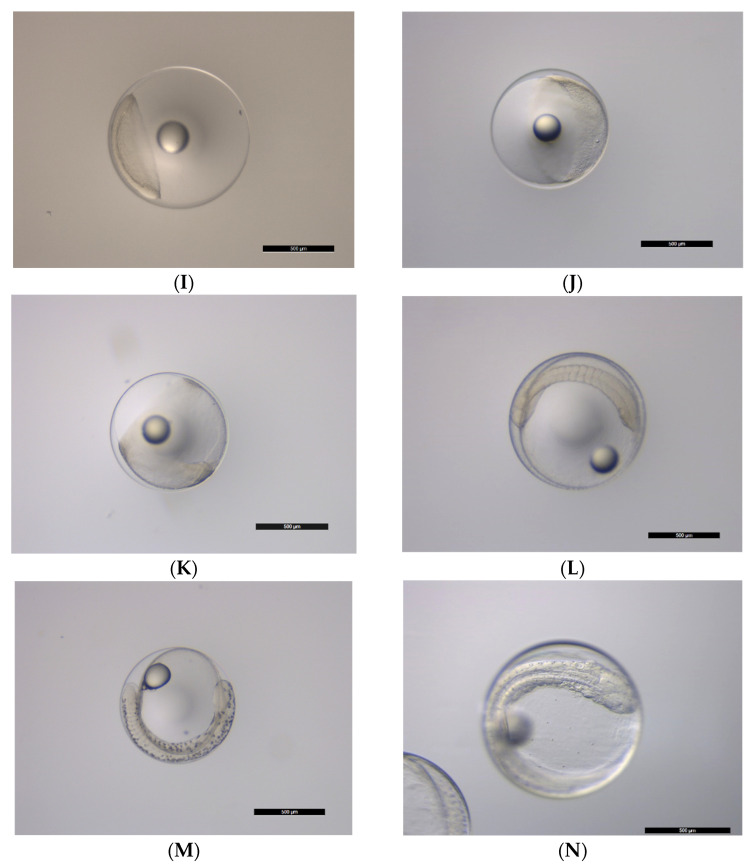
Embryonic development of *Chaetodontoplus mesoleucus*. (**A**) Fertilized egg; (**B**) 2-cell stage with a blastomere; (**C**) 4-cell stage; (**D**) 8-cell stage; (**E**) 16-cell stage; (**F**) 32-cell stage; (**G**) 64-cell stage; (**H**) Morula stage; (**I**) Blastula stage; (**J**) 30% epiboly completion; (**K**) Gastrula stage: 70% epiboly completion; (**L**) Neurula stage; (**M**) melanophores occurring on embryo; (**N**) melanophores occurring on embryo, oil globule, and yolk, heartbeat stage with otic vesicles. The scale bar = 500 μm.

The fertilized eggs of *C. mesoleucus* are spherical, transparent, and buoyant, containing a single oil globule. The key characteristics of embryonic development are summarized in Table 1. Under controlled conditions of 27 ± 0.5 °C and a salinity of 33.5 ± 0.5 psu, the fertilized eggs diameters ranged from 0.90 to 0.96 mm (0.93 ± 0.02 mm, *n* = 15), while the oil globule diameter varied between 0.22 and 0.23 mm (0.23 ± 0.01 mm, *n* = 15) (Figure 1A). The egg reached the 2-cell stage at 40 min post-fertilization (pf), showing the first cleavage (Figure 1B). The 4-, 8-, 16-, 32-, and 64-cell stage were reached at 50 min pf, 1 h 2 min pf, 1 h 14 min pf, 1 h 27 min pf, and 1 h 38 min pf, respectively (Figure 1C–G). The embryo reached the high stage at 1 h 58 min pf, at which time the blastomeres were countless (Figure 1H). At 7 h 10 min pf, the blastodisc covered approximately 30% of the yolk (Figure 1J). By 8 h 3 min pf, the gastrula stage commenced, with the embryo covering 70% of the yolk, and the embryonic shield appearing (Figure 1K). At 8 h 55 min pf, the tail bud appeared, and the blastopore closed, reaching the neurula stage (Figure 1L). At 9 h 38 min pf, the myomeres and optic lens appeared. At 10 h 12 min pf, melanophores appeared on the body surface (Figure 1M). At 15 h 48 min pf, the heart and auditory vesicles appeared. At 21 h and 55 min pf, embryonic movements became more pronounced, and hatching commenced, with larvae emerging head-first from the egg membrane (Figure 1N).

### 3.2. Yolk Sac and Oil Globule Utilization

**Figure 2:**
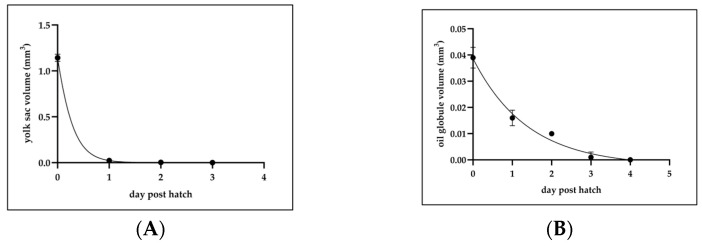
(**A**)Yolk sac and (**B**) oil globule utilization pattern of *C. mesoleucus* yolk-sac larvae (*n* = 10). All data were expressed as means ± SD.

The yolk sac and oil globule utilization patterns of *C. mesoleucus* larvae were analyzed from hatching to 3 dph. At hatching, the yolk sac volume ranged from 0.98 to 1.40 mm^3^ (1.14 ± 0.07 mm^3^, *n* = 10). At 1 dph, the yolk sac volume decreased to 2–4 × 10^−2^ mm^3^ (2.3 ± 0.7 × 10^−2^ mm^3^, *n* = 10). At 2 dph, the yolk sac volume further declined to 0.1–5.5 × 10^−3^ mm^3^ (3 ± 2 × 10^−3^ mm^3^, *n* = 10). The quadratic equation describing yolk sac utilization was derived as follows: y = 0.8368e^−2.971x^, R^2^ = 0.9992, where y represents the yolk sac volume (mm^3^) and x represents dph (Figure 2A). The oil globule volume at hatching ranged from 3.0 to 4.0 × 10^−2^ mm^3^ (3.9 ± 0.4 × 10^−2^ mm^3^, *n* = 10). At 1 dph, it decreased to 1.0–2.0 × 10^−2^ mm^3^ (2.3 ± 0.7 × 10^−2^ mm^3^, *n* = 10). At 2 dph, the oil globule volume was 0.0–1.0 × 10^−2^ mm^3^ (1.0 ± 0.1 × 10^−2^ mm^3^, *n* = 10), and by 3 dph it was further reduced to 0.0–1.0 × 10^−2^ mm^3^ (1.0 ± 2 × 10^−3^ mm^3^, *n* = 10). The quadratic equation describing oil globule utilization was the following: y = 0.0496e^−1.146x^, R^2^ = 0.971, where y represents the oil globule volume (mm^3^) and x represents dph (Figure 2B).

### 3.3. Development of Larvae and Juveniles

**Figure 3:**
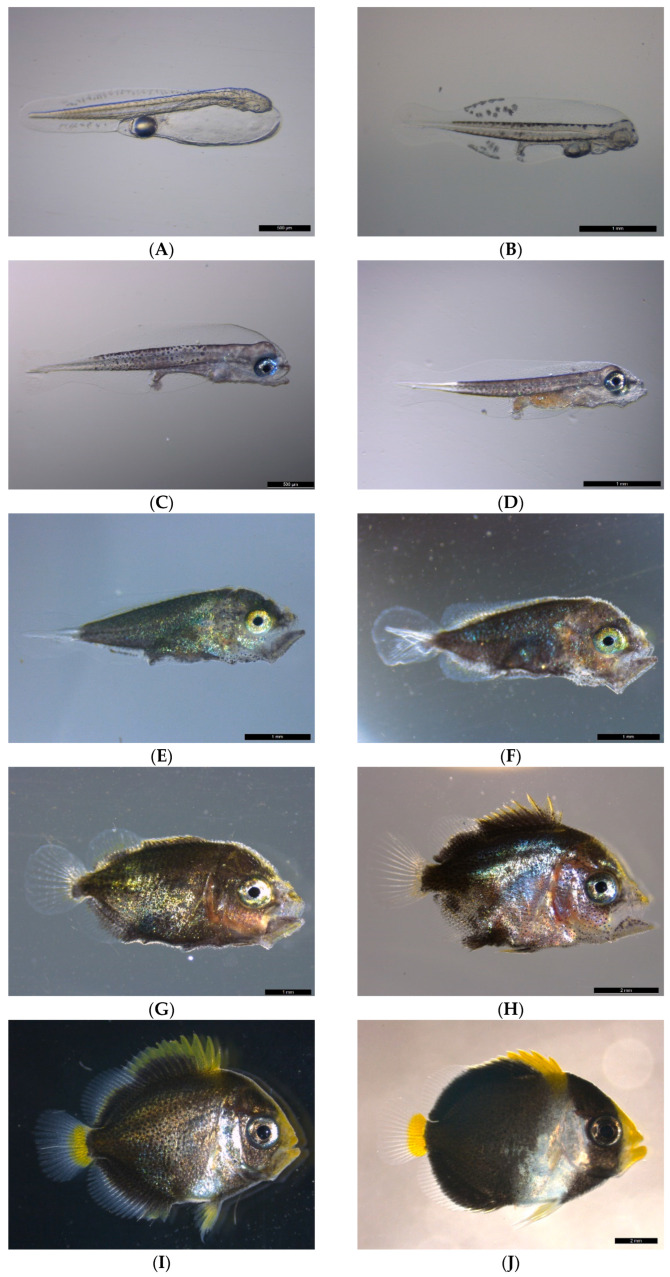
Larval development of *C. mesoleucus*. (**A**) Newly hatched yolk-sac larva; (**B**) 1 dph yolk-sac larva; (**C**) 2 dph yolk-sac larva; (**D**) 3 dph larva with functional mouth that begins feeding; (**E**) 6 dph preflexion larva; (**F**) 9 dph flexion larva; (**G**) 12 dph postflexion larva; (**H**) 19 dph transformed larva; (**I**) 28 dph juveniles; (**J**) 32 dph juveniles with full coloration as broodstock is apparent. Scale bar of (**A**,**C**) = 500 μm. Scale bar of (**B**,**D**–**G**) = 1 mm. Scale bar of (**H**,**J**) = 2 mm.

The key characteristics of larvae and juveniles are summarized in Table 2. Newly hatched larvae measured 2.34 to 2.55 mm in TL (2.50 ± 0.07 mm, *n* = 10). A single oil globule was positioned at the posterior end of the yolk sac. Melanophores were distributed along the dorsal and ventral finfold edges. The myomere count ranged from 26 to 27 (11–13 + 12–15) pairs. At this stage, the eyes, mouth, and digestive system were underdeveloped, and the larvae relied on yolk sac and oil globule reserves for nutrition (Figure 3A). At 1-day post-hatching (1 dph), the larvae grew to 3.29-3.42 mm TL (3.32 ± 0.06 mm, *n* = 10). The yolk sac exhibited a significant reduction in size, while the oil globule was partially consumed. The number of stellate melanophores increased, particularly along the myomere boundaries, head, and finfolds near the vent. The eyes were unpigmented, and the intestine extended to approximately half their body length (Figure 3B). At 2 dph, the larvae measured 3.23–3.39 mm in TL (3.31 ± 0.05 mm, *n* = 10). The yolk sac was nearly depleted, and the oil globule volume was reduced by approximately 50%. Melanophores were uniformly distributed along the myomeres, except for the caudal region, where pigmentation was absent. The eyes, mouth, and digestive tract were beginning development, and a few larvae contained food particles in their intestines, indicating the initiation of exogenous feeding (Figure 3C). At 3 dph, the larvae measured 3.06–3.37 mm in TL (3.26 ± 0.10 mm, *n* = 10). The oil globule was nearly depleted, and the melanophores were densely covered the myomeres, except in the caudal region. Swimming and feeding activity increased, and the anus opened, with the digestive tract consistently filled with food (Figure 3D). At 6 dph, the larvae grew to 4.17–4.48 mm TL (4.31 ± 0.13 mm, *n* = 5). Melanophore density increased, giving the larvae a darker appearance. Spine formation began on the head, and the swim bladder started to inflate, marking the transition to the preflexion stage (Figure 3E). At 8 dph, the larvae reached 4.31-5.08 mm in TL (4.60 ± 0.31 mm, *n* = 5), with dorsal, anal, and caudal fin development initiated. At 9 dph, the larvae measured 4.31-5.08 mm in TL (4.61 ± 0.30 mm, *n* = 5). The body deepened, melanophores extended to the dorsal and anal fin bases, and xanthophores appeared, indicating the flexion stage (Figure 3F). At 12 dph, the larvae grew to 4.85–5.67 mm in TL (5.25 ± 0.37 mm, *n* = 10). The body continued to elongate, and yellow-green metallic sheen was observed in many larvae. Yellow chromatophores appeared on the dorsal, anal, and caudal fins. Dorsal fin rays counted XI/16, and anal fin rays counted I–II/15, marking the postflexion stage (Figure 3G). At 19 dph, the larvae reached 7.27–9.02 mm in TL (7.71 ± 0.51 mm, *n* = 10). A silvery metallic sheen developed in the pectoral fin area, and yellow pigmentation became prominent on the head, dorsal fin, and caudal peduncle (Figure 3H). At 28 dph, the juveniles measured 9.86–10.71 mm in TL (10.32 ± 0.31 mm, *n* = 5). The overall fin ray counts attained an adult complement: dorsal fin rays counted XII/18, and anal rays counted III/18. The dorsal fin, pelvic fins, and the base of the caudal fin of the fry were starting to turn yellow, and the edge of the operculum extending to the area around the pectoral fins was beginning to turn white (Figure 3I). At 32 dph, the juveniles reached 12.00–13.12 mm in TL (12.50 ± 0.44 mm, *n* = 5), exhibiting the exact same body coloration as the broodstock. At 62 dph, the juveniles measured 16.96–19.67 mm in TL (18.47 ± 1.16 mm, *n* = 8), and the colors became more vibrant. Approximately 30% of the juveniles swam to the bottom of the tank and began seeking shelter, while the remaining individuals remained near the water surface. A small amount of artificial feed was initially introduced, followed by a gradual reduction in the density of live prey and a corresponding increase in the quantity of artificial feed. (Figure 3J). At 89 dph, the juveniles reached 18.75–23.76 mm in TL (22.06 ± 1.59 mm, *n* = 8); they were now able to start feeding on artificial feed. All individuals moved toward the bottom of the tank and exhibited increased activity, including competing for shelter. Additional coral stones were subsequently provided to increase the available hiding spaces. At 381 dph, the juveniles attained 49.55–71.25 mm in TL (62.37 ± 7.62 mm, *n* = 6); all the juveniles were transferred to the recirculating aquaculture system, where they exhibited schooling behavior and tended to feed and swim in groups. 

**Figure 4:**
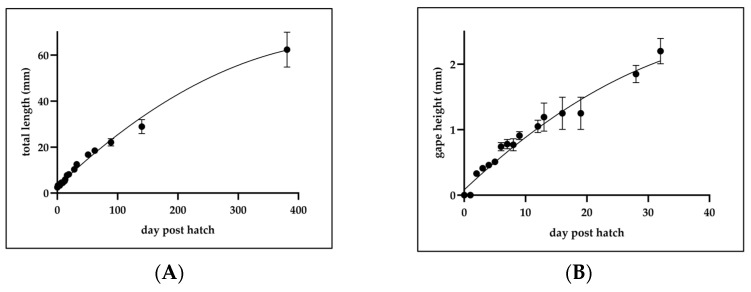
Morphometric development of *C. mesoleucus* larvae. (**A**) TL (mm) (*n* > 5) and (**B**) gape height (mm) (*n* = 10). All data were expressed as means ± SD.

At 2 dph, the gape height was 33 ± 3.6 × 10^−2^ mm (*n* = 10). At 3 dph, it increased to 41.1 ± 2.4 × 10^−2^ mm (*n* = 10). At 9 dph, the gape height reached 9.1 ± 0.6 × 10^−1^ mm (*n* = 4), and at 12 dph, it measured 10.5 ± 0.69 × 10^−1^ mm (*n* = 10). The quadratic growth equation for gape height was determined as follows: y = −0.0007x ^2^ + 0.0847x + 0.1702, R^2^ = 0.9844, where y represents gape height (mm) and x represents dph (Figure 4A).

The growth trend of *C. mesoleucus* larvae shows slow growth initially, but it starts to accelerate significantly after 13 dph. The quadratic growth equation for TL over 381 dph was determined as follows: y = −0.0013x^2^ + 0.3452x + 2.1334, R^2^ = 0.9936, where y represents TL (mm) and x represents dph (Figure 4B).

### 3.4. Effects of Temperature on Hatch Rate, Time to 50% Hatch, Hatching Period Duration, and Deformity Rate

**Figure 5:**
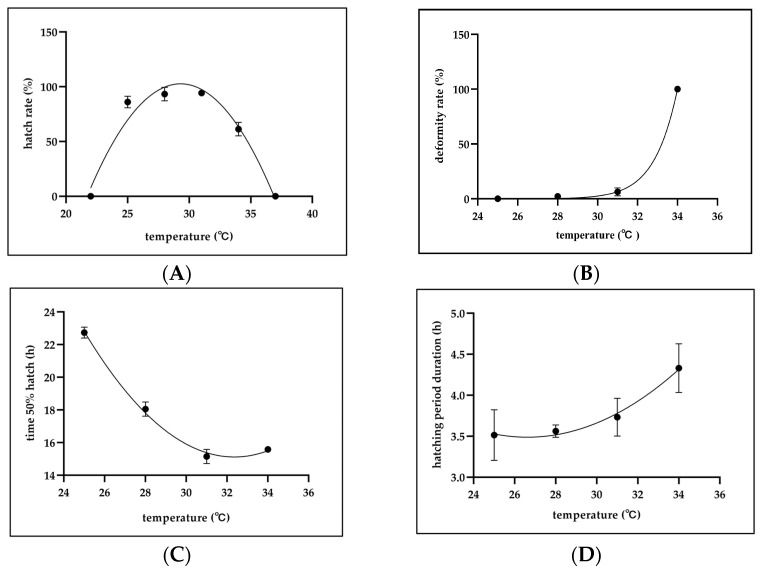
Effects of temperature on hatch rate, deformity rate, time to 50% hatch, and hatching period duration on *C. mesoleucus* embryos. (**A**) Mean hatch rate (%) of the embryos (*n* = 50); (**B**) mean deformity rate (%) of the larvae (*n* = 50); (**C**) mean time to 50% hatch (h) of the embryos (*n* = 50); (**D**) mean hatching period duration (h) of the embryos (*n* = 50).All treatments were performed in triplicate. Data are expressed as means ± SD.

At 25 °C, 28 °C, and 31 °C, the mean hatch rates were 86.0 ± 5.3%, 93.3 ± 6.1%, and 94.0 ± 2.0%, respectively, which were significantly higher than 34 °C (*p* < 0.05). A notable decline was observed at 34 °C (61.3 ± 6.1%; *p* < 0.05), while no hatching occurred at either 22 °C and 37 °C, representing a significant reduction compared to the others (*p* < 0.05) (Figure 5A). The deformity rate of the larvae increased with temperature. At 34 °C, the deformity rate reached 100.0 ± 0.0%, significantly higher than in all other treatments (*p* < 0.05). At 31 °C, the deformity rate was 27.0 ± 1.4%, which was significantly higher than the 0.0% recorded at 25 °C (*p* < 0.05). At 28 °C, the rate was 1.6 ± 1.4%, which was not significantly different from the rates observed in the 25 °C and 31 °C groups (*p* > 0.05) (Figure 5B). The time that elapsed for 50% hatching was negatively correlated with the temperature. At 25 °C, the time to 50% hatch was the longest, recorded at 22.7 ± 0.3 h pf, which was significantly higher than other treatments (*p* < 0.05). At 28 °C, the time to 50% hatch was longest, recorded at 18.01 ± 0.5 h, which was significantly than other treatments (*p* < 0.05). At 31 °C and 34 °C, the 50% hatching times were 15.15 ± 0.4 h, and 15.6 ± 0.2 h pf, respectively; these values were not significantly different from each other (*p* > 0.05), but both differed significantly from those at other temperatures. (*p* < 0.05). No hatch was observed at 22 °C and 37 °C. (Figure 5C). The hatching period duration increased with the temperature. The mean hatching duration at 34 °C was 4.3 ± 0.3 h pf, which was significantly longer than those at 25 °C (3.5 ± 0.3 h) and 28 °C (3.6 ± 0.1 h) (*p* < 0.05). The duration at 31 °C (3.7 ± 0.2 h) was not significantly different from the other groups (*p* > 0.05) (Figure 5D).

**Figure 6:**
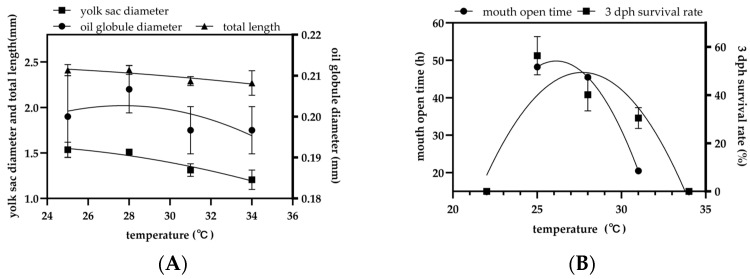
Effects of temperature on (**A**) 0 dph TL (mm), yolk-sac diameter (mm), globule diameter (mm) (*n* = 10), (**B**) mouth open time (*n* = 10), and 3 dph survival rate (%) (*n* = 50) of *C. mesoleucus* larvae.

The yolk-sac diameter of the newly hatched larvae was negatively correlated with the incubation temperature. At 25 °C, yolk-sac length was 1.54 ± 0.84 mm, which was significantly greater than at 31 °C (1.31 ± 0.07 mm) and 34 °C (1.21 ± 0.11 mm) (*p* < 0.05). The larvae reared at 28 °C had a yolk-sac length of 1.51 ± 0.03 mm, which was not significantly different from those at 25 °C or 31 °C (*p* > 0.05), but remained significantly larger than those at 34 °C (*p* < 0.05). There was no significant difference between the 31 °C and 34 °C groups (*p* > 0.05). The oil globule diameter of the newly hatched larvae was highest at 28 °C, measuring 0.21 ± 0.01 mm, which was significantly larger than the diameters observed at 25 °C, 31 °C, and 34 °C (each 0.20 ± 0.01 mm; *p* < 0.05). Larval TL at hatching also tended to decrease with higher temperatures, although the differences in TL among the temperature treatments were not statistically significant (*p* > 0.05) (Figure 6A).

At 3 dph, the survival rate at 25 °C was 56.4 ± 7.9%, which was significantly higher than all other temperature groups (*p* < 0.05). The 28 °C and 31 °C groups showed survival rates of 40.2 ± 6.7% and 30.5 ± 4.3%, respectively, with no significant difference between these two treatments (*p* > 0.05). No larvae survived at 22 °C or 34 °C (0.0%), which was a significantly lower amount than in all other treatments (*p* < 0.05). The time to mouth opening in the larvae was inversely related to the temperature. The larvae reared at 25 °C began feeding (opened their mouths) at approximately 48.3 h post-hatch, those at 28 °C began at 45.5 h, and those at 31 °C began as early as 20.5 h post-hatch. All larvae in the 22 °C and 34 °C treatment groups died before reaching the mouth-opening stage, so no mouth-opening time could be recorded for these groups (Figure 6B).

### 3.5. Effects of Salinity on Hatch Rate and Deformity Rate

**Figure 7:**
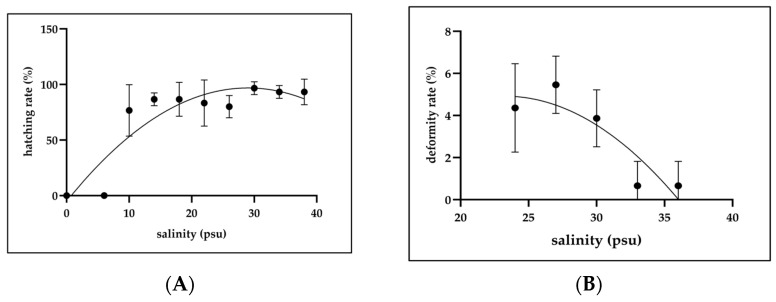
Effect of salinity on (**A**) hatch rate (%) (*n* = 10) and (**B**) deformity rate (%) (*n* = 50). All treatments were performed in triplicate. Data are expressed as means ± SD.

The fertilized eggs were incubated under a range of salinity conditions from 0 to 38 psu. Hatch success was the highest at 30 psu (96.7 ± 5.8%) and remained high at 34 psu (93.3 ± 5.8%) and 38 psu (93.3 ± 11.6%). Hatch rates at 10 to 26 psu ranged from 76.7 ± 23.1% to 86.7 ± 15.28%, with no significant differences among these treatments (*p* > 0.05). However, hatching was completely inhibited at 0 and 6 psu (0.0%), which was a significantly lower amount than in the other groups (*p* < 0.05). The quadratic regression for hatch rate as a function of salinity was the following: y = −0.0012x^2^ + 0.0704x − 0.0521, R^2^ = 0.8148, where *y* is the hatch rate (%) and *x* is salinity (psu) (Figure 7A). Deformity rates varied with salinity. The highest deformity rate was recorded at 27 psu (5.3 ± 1.5%), followed by 24 psu (4.4 ± 2.1%) and 30 psu (3.8 ± 1.3%). These values were not significantly different (*p* > 0.05). However, the deformity rates at 33 and 36 psu were significantly lower (0.8 ± 1.3% and 0.8 ± 1.4%, respectively; *p* < 0.05). The quadratic equation representing the relationship between salinity and deformity rate was as follows: y = −0.0003x^2^ + 0.0124x − 0.0914, R^2^ = 0.8103, with *y* denoting deformity rate (%) and *x* representing salinity (psu) (Figure 7B).

## 4. Discussion

### 4.1. Early Development

This study significantly advances our understanding of the early developmental stages of *C. mesoleucus*, providing a detailed account that extends considerably beyond the previously documented 68 hph [10]. While comprehensive developmental data exists for the congener *C. septentrionalis* up to 137 dph [25], our knowledge of *C. mesoleucus* [10] and *C. duboulayi* [26] development has historically been limited to the initial two to three days. The extended observations presented here thus fill a critical knowledge gap, offering crucial insights into the specific requirements and developmental trajectory of *C. mesoleucus* during its early life. This enhanced dataset enables a more robust comparative analysis of key developmental milestones and morphometrics within the *Chaetodontoplus* genus, which is essential for identifying the factors that are critical for initial survival and successful aquaculture.

Key early developmental metrics, including egg size and initial larval length, exhibited notable variations in our study compared to previous reports on *C. mesoleucus* and also among related *Chaetodontoplus* species. We recorded larger fertilized egg diameters (0.93 ± 0.02 mm) and newly hatched larval TLs (2.50 ± 0.07 mm) for *C. mesoleucus* than those reported by [10] (0.82–0.88 mm eggs; 1.9–2.0 mm larvae). Interestingly, our measured egg size was larger than that of the larger-bodied *C. septentrionalis* (0.83 ± 0.02 mm; [25]) but comparable to *C. duboulayi* (0.92–0.97 mm; [26]). However, the hatching time observed in our study (21 h 55 min at 27 ± 0.5 °C) was consistent with the findings in [10] when accounting for potential temperature differences, and the sizes of oil globules were broadly similar across studies. These discrepancies in egg and larval size could potentially arise from variations in broodstock populations, environmental conditions under which the broodstock were maintained, or subtle differences in the experimental methodologies employed. Establishing these updated baseline metrics for *C. mesoleucus* under current controlled conditions is therefore vital for future comparative studies and for identifying the specific factors that contribute to early developmental success in aquaculture settings.

Our findings strongly suggest that variations in egg quality, likely influenced by broodstock condition and maturity, play a significant role in determining early larval size, subsequent development, and overall survival prospects in *C. mesoleucus*. The larger egg and subsequent larval sizes observed in our study coincided with successful rearing beyond the 68 hph mark, a stark contrast to [10], where complete larval mortality occurred after this point. Notably, we observed smaller eggs (0.86–0.88 mm) from the early spawning females during our trials, suggesting a potential maturity effect on egg quality, which aligns with findings in other fish species where broodstock status significantly impacts egg size [27,28]. The literature widely supports the notion that a larger egg size often correlates positively with enhanced larval survival and overall rearing success across various fish species [27,29]. This trend is also reflected interspecifically within the *Chaetodontoplus* genus, with *C. septentrionalis*, which has the smallest eggs and larvae [25,30]; there is also a relatively high level of difficulty in larval rearing. Furthermore, the timely development of functional structures crucial for first feeding, such as a mouth gape (41.1 ± 2.4 × 10^−2^ mm at 3 dph in *C. mesoleucus*), also appears to be linked to initial egg and larval size, potentially providing larvae hatched from larger eggs with a developmental advantage. Consequently, the superior egg size observed in our study likely conferred critical advantages in initial larval size and overall robustness, contributing significantly to their successful survival beyond the previously documented bottleneck. These findings underscore the factors influencing egg quality, particularly broodstock management and maturity, that are paramount for optimizing early developmental outcomes and enhancing survival rates in *C. mesoleucus* aquaculture.

### 4.2. Effects of Temperature on Hatching and Larval Development

Incubation temperature exerts a profound influence on both the success of hatching and, critically, the quality of the resulting *C. mesoleucus* larvae, demonstrating that solely evaluating hatching rate provides an incomplete assessment of developmental success. Our study revealed that, while hatching rates remained relatively high across a temperature range from 22 °C to 37 °C, the biological outcomes differed markedly at the upper extreme. Specifically, incubation at 31 °C led to a severe increase in larval deformities, affecting nearly all the hatchlings observed. In stark contrast, eggs incubated at 25 °C and 28 °C not only achieved high hatching success but also yielded larvae with exceptionally low deformity rates (0.0% and 1.6 ± 1.4%, respectively), indicating robust development within this temperature range.

This distinct pattern strongly suggests that, although *C. mesoleucus* embryos possess the physiological capacity to complete the hatching process at 31 °C, this temperature significantly exceeds the species’ optimal thermal window for normal morphogenesis, resulting in the induction of severe and pervasive malformations. This finding underscores a crucial point frequently encountered in fish larviculture: maximizing hatching quantity is counterproductive if larval quality and subsequent viability are significantly compromised. Indeed, parallel observations exist in other species; for instance, studies on the tropical snapper *Lutjanus carponotatus* [31] and the temperate lingcod *Ophiodon elongatus* [32] similarly report that supra-optimal incubation temperatures can yield superficially high hatch numbers but result in non-viable or severely impaired larvae due to accumulated developmental stress and potentially accelerated, inefficient yolk consumption [33,34,35]. In all the above studies, warmer-than-optimal temperatures tended to shorten the incubation period but at the cost of reduced hatching success and increased malformation or deformity rates in the larvae. Cooler incubation often produced healthier, larger hatchlings but took longer and, if it is too cold, this could reduce hatching success [33,35]. Therefore, a comprehensive assessment strategy—incorporating a detailed evaluation of larval morphology and viability alongside traditional hatching percentages—is imperative for identifying truly optimal incubation conditions, which our data robustly place within the 25–28 °C range for ensuring the production of healthy *C. mesoleucus* larvae.

The experimentally determined optimal incubation range of 25–28 °C for *C. mesoleucus* shows strong congruence with the natural thermal habitat of the species; yet, it also reveals an unusual hatching response at supra-optimal temperatures compared to the generally observed patterns in teleosts. This optimal laboratory range favorably overlaps with the species’ preferred environmental temperatures, documented in the wild (25.5–29 °C; [36]). Such alignment lends support to the broader ecological principle that fish species often synchronize their reproduction with the thermal conditions that maximize embryonic survival and successful development [37]. Typically, higher incubation temperatures lead to shorter hatching period durations and thus faster hatching. Studies on Walleye pollock (*Gadus chalcogrammus*), Atlantic cod (*Gadus morhua*), and clownfish (*Amphiprion ocellaris*) have all demonstrated a clear negative correlation between incubation temperature and hatching period duration [38,39,40]. This general trend—wherein an elevated temperature shortens the duration of the hatching period—has been confirmed by many researchers [17].

Furthermore, we noted that many of the embryos hatching significantly later at 31 °C were malformed. This suggests that these developmentally compromised individuals struggled to rupture the chorion and emerge successfully. This abnormal delay implies that extreme thermal stress imposes a significant physiological burden that ultimately hinders, rather than accelerates, the completion of the hatching process—particularly for embryos already weakened by suboptimal conditions.

Integrating the comprehensive findings on hatching rates, larval deformity prevalence, and hatching dynamics provides a clear and robust basis for recommending the optimal incubation temperatures essential for the successful and efficient hatchery production of *C. mesoleucus*. The collective data unequivocally underscore that the 25–28 °C range uniquely balances high hatching success with the crucial production of morphologically normal, healthy larvae exhibiting synchronous hatching.

### 4.3. Effects of Salinity on Hatch Rate and Deformity Rate

Our study demonstrates that *C. mesoleucus* embryos possess a distinct lower salinity threshold that is essential for successful hatching—a physiological trait commonly observed in many marine teleost species. Normal hatching readily occurred at salinities above 10 psu, whereas hatching success dropped to zero below this critical level. This result aligns with our unpublish findings for the congener *C. septentrionalis*, which also failed to hatch at salinities below 10 psu. Such abrupt declines in hatching success below species-specific salinity thresholds, and optimal hatching being restricted to a relatively narrow range close to natural seawater value, are well documented across a wide range of marine teleosts, including Pacific herring (*Clupea pallasii*) [41], black porgy (*Oplegnathus fasciatus*), and Chinese silver pomfret (*Pampus punctatissimus*) [22,42].

Among the marine fishes with pelagic eggs, one common consequence of low salinity is reduced egg buoyancy, which can cause eggs or yolk-sac larvae to sink below the optimal environmental strata [43]. Although marine reef fish eggs are generally capable of hatching at salinities moderately below full-strength seawater, and larvae can survive and grow under such conditions for extended periods [3,44,45], extremely low salinities—approaching freshwater—have been shown to result in high mortality and increased incidence of developmental abnormalities [3]. In the early developmental stages, embryos are not yet capable of active osmoregulation and instead depend on the egg’s structural and biochemical adaptations to cope with external osmotic pressure [46]. When ambient salinity falls below a tolerable threshold, osmotic imbalance may lead to excessive water influx, resulting in egg swelling, edema, or even a rupture of the egg membrane. Thus, there exists a lower salinity limit below which reef fish embryos and larvae are unable to maintain osmotic homeostasis.

In species such as seabass, rearing larvae in excessively low salinities can disrupt normal swim bladder inflation and lead to skeletal deformities such as lordosis, due to buoyancy-related stress [47]. Conversely, rearing marine embryos at salinities higher than their physiological optimum has also been associated with increased frequencies of craniofacial and spinal deformities [48]. This inherent sensitivity to salinity underscores the potential developmental risks posed by natural salinity fluctuations, such as those resulting from heavy rainfall, freshwater influxes in estuarine areas, or evaporation-driven salinity spikes in shallow coastal environments [49].

Therefore, defining the lower salinity limit (10 psu) for *C. mesoleucus* is crucial for understanding this species’ environmental tolerance range and for the development of effective hatchery protocols. Maintaining stable salinity conditions near natural seawater levels is essential not only for optimizing hatching success but, more importantly, for ensuring the production of morphologically normal, high-quality larvae that are critical for subsequent survival and successful rearing.

## 5. Conclusions

In conclusion, this study provides a comprehensive advancement in our understanding of the early life history of *Chaetodontoplus mesoleucus*. Our detailed ontogenetic observations significantly extend the known developmental timeline for this species, revealing larger egg and larval sizes than previously reported and highlighting the critical influence of egg quality, likely determined by broodstock condition, on early larval success. Furthermore, we have precisely defined the optimal thermal window for incubation, identifying 25–28 °C as the range that maximizes hatching success while ensuring the production of high-quality, morphologically normal larvae. The detrimental effects of exceeding this range, particularly the severe deformities observed at 31 °C and the associated disruption of the hatching process, underscore the importance of careful temperature management in *C. mesoleucus* aquaculture. Finally, our investigation into salinity tolerance has established a lower hatching threshold of 10 psu and an optimal range of 33–36 psu for minimizing larval deformities, emphasizing the necessity of maintaining stable, near-natural salinity levels for successful larval development. These findings collectively provide crucial baseline data and practical recommendations for optimizing hatchery protocols for *C. mesoleucus*, ultimately contributing to the enhanced survival and successful rearing of this ornamental fish species. Future research could further explore the specific factors influencing broodstock egg quality and investigate the long-term effects of the identified optimal environmental parameters on growth and development beyond the larval stage.

## Figures and Tables

**Table 1:** Key morphological characteristics at the embryonic developmental stage of *C. mesoleucus*, cultured at 26.7 ± 0.3 °C.

Developmental Stage	Duration Time	Key Morphological Characteristic
Zygote period		
Fertilized egg		Newly fertilized; blastodisc appears; one oil globule; 0.93 ± 0.02 mm in diameter
Cleavage period		
2-cell	40 min pf	1st cleavage; dividing the blastodisc into 2 blastomeres
4-cell	50 min pf	2nd cleavage; perpendicular to the first
8-cell	1 h 2 min pf	3rd cleavage
16-cell	1 h 14 min pf	4th cleavage
32-cell	1 h 27 min pf	5th cleavage
64-cell	1 h 38 min pf	6th cleavage
Blastula period		
High	1 h 58 min pf	Epiboly process began
30%-epiboly	7 h 10 min pf	Epiboly comes to 30%
Gastrula		
70%-epiboly	8 h 3 min pf	Epiboly comes to 70%; embryonic shield appears; differentiation of embryonic axis occurs
Embryonic development		
Neurula	8 h 55 min pf	Blastopore closes; tail bud appears
Embryo	9 h 38 min pf	Myomeres and optical lens appear
	10 h 12 min pf	Melanophores occur on embryo; otic vesicle appears
	15 h 48 min pf	Heart is discernible; auditory vesicles are developed
Before hatching	21 h 50 min pf	Tail separates from yolk and spins freely
Free yolk-sac larvae	21 h 55 min pf	Hatching begins; larva is free from the membrane

pf, post fertilization.

**Table 2:** Key morphological characteristics at each developmental stage of *C. mesoleucus* larvae and juveniles; cultured at the temperature 27 ± 0.5 °C.

Developmental Stage	Duration Time	Key Morphological Characteristic
Larval period		
Yolk-sac	0 dph	26 myomeres; one big yolk sac; 2.50 ± 0.07 mm *L*_T_
	1 dph	Alimentary canal appears but is not yet functional; yolk reduces in size; melanophores scatter along the entire body and the dorsal and ventral finfolds behind the anus; 3.32 ± 0.06 mm *L*_T_
	2 dph	Mouth opening appears but is not yet functional; melanophores scatter along the entire body; eyes, mouth and digestive tract were in development; 3.31 ± 0.05 mm *L*_T_
	3 dph	Yolk is completely absorbed; functional mouth appears and begins feeding; 3.26 ± 0.10 mm *L*_T_
Preflexion	6 dph	Body becomes deeper; swim bladder appears; short spinules begin to form on the head; 4.31 ± 0.13 mm *L*_T_
	8 dph	Soft rays of the dorsal and anal fins appear; 4.60 ± 0.31 mm *L*_T_
Flexion	9 dph	Notochord end becomes flexed; 4.61 ± 0.30 mm *L*_T_
Postflexion	12 dph	Hypural bones assume a vertical position; 5.25 ± 0.37 mm *L*_T_
Transformation	19 dph	Yellow areas appear above the head and base of the caudal fin; pelvic fin spines appear; 7.71 ± 0.51 mm *L*_T_
Juvenile period		
Juvenile	28 dph	Fin ray counts attain an adult complement; 10.32 ± 0.31 mm *L*_T_
	32 dph	Full juvenile coloration is apparent with white area in the middle, and yellow areas appearing on the mouth, tail, caudal fin, dorsal fin, and pectoral fin; 12.50 ± 0.44 mm mm *L*_T_

*L*_T_, total length; dph, days post hatch.

## Data Availability

Data are contained within the article.

## Supplementary Materials

The following supporting information can be downloaded at https://www.mdpi.com/article/10.3390/ani15111657/s1. Table S1: Effects of temperature on hatch rate (%) of the embryos. Table S2: Effects of temperature on deformity rate (%) of the larvae. Table S3: Effects of temperature on mean time to 50% hatch (h) of the embryos. Table S4: Effects of temperature on mean hatching period duration (h) of the embryos. Table S5: Effects of temperature on 3 dph survival rate (%) of the larvae. Table S6: Effect of salinity on hatch rate (%) of the embryos. Table S7: Effect of salinity on deformity rate (%) of the larvae.
