# Supplementary material for: Natural Spawning, Early Development, and First Successful Hatchery Production of the Vermiculated Angelfish (Chaetodontoplus mesoleucus), Exploring the Influence of Temperature and Salinity
Source: Animals (Basel). 2025 Jun 4;15(11):1657. doi: 10.3390/ani15111657 (PMC12153641; doi:10.3390/ani15111657)
Supplement: Supplementary file 1 [file animals-15-01657-s001.zip › animals-3621624-supplementary.pdf]

Supplementary Table

Table S1. Effects of temperature on hatch rate (%) of the embryos.

**Temp**	**22℃**	**25℃**	**28℃**	**31℃**	**34℃**	**37℃**
**Minimum**	0	82.0	88.0	90.0	56.0	0
**Maximum**	0	92.0	100.0	94.0	68.0	0
**Mean**	0	86.0	93.3	92.0	61.3	0
**SD**	0	5.29	6.11	2.00	6.11	0
**SE**	0	3.06	3.53	1.16	3.53	0
**Variance**	0	28.01	37.33	4.00	37.33	0
**F (DFn, DFd)**	F (5, 12) = 345.7					
**P value**	P<0.0001					

Table S2. Effects of temperature on deformity rate (%) of the larvae.

**Temp**	**22**	**25**	**28**	**31**	**34**	**37**
**Minimum**	0	0	2.0	2.6	100.0	0
**Maximum**	0	0	2.5	9.8	100.0	0
**Mean**	0	0	2.2	6.3	100.0	0
**SD**	0	0	0.25	3.60	0	0
**SE**	0	0	0.15	2.08	0	0
**Variance**	0	0	0.06	12.99	0	0
**F (DFn, DFd)**	F (3, 8) = 2176					
**P value**	P<0.0001					

Table S3. Effects of temperature on mean time to 50% hatch (h) of the embryos

**Temp**	**22**	**25**	**28**	**31**	**34**	**37**
**Minimum**	0	22.5	17.8	14.7	15.4	0
**Maximum**	0	23.1	18.6	15.5	15.8	0
**Mean**	0	22.7	18.1	15.2	15.6	0
**SD**	0	0.34	0.44	0.44	0.20	0
**SE**	0	0.19	0.25	0.25	0.11	0
**Variance**	0	0.12	0.19	0.19	0.04	0
**F (DFn, DFd)**	F (3, 8) = 271.5					
**P value**	P<0.0001					

Table S4. Effects of temperature on mean hatching period duration (h) of the embryos.

Temp	22	25	28	31	34	37
**Minimum**	0	3.2	3.5	3.6	4.1	0
**Maximum**	0	3.7	3.6	4.0	4.7	0
**Mean**	0	3.51	3.56	3.74	4.33	0
**SD**	0	0.31	0.08	0.22	0.30	0
**SE**	0	0.18	0.04	0.13	0.17	0
**Variance**	0	0.10	0.01	0.05	0.09	0
**F (DFn, DFd)**	F (3, 8) = 7.057					
**P value**	P=0.0123					

Table S5. Effects of temperature on 3 dph survival rate (%) of the larvae.

Temp	22	25	28	31	34	37
**Minimum**	0	47.6	34.8	25.5	0	0
**Maximum**	0	63.0	47.7	33.3	0	0
**Mean**	0	56.7	40.2	30.5	0	0
**SD**	0	8.07	6.72	4.32	0	0
**SE**	0	4.66	3.88	2.49	0	0
**Variance**	0	65.17	45.12	18.62	0	0
**F (DFn, DFd)**	F (5, 12) = 85.29					
**P value**	P<0.0001					

Table S6. Effect of salinity on hatch rate (%) of the embryos.

**Salinity**	**0**	**6**	**10**	**14**	**18**	**22**	**26**	**30**	**34**	**38**
**Minimum**	0	0	50.0	80.0	70.0	60.0	70.0	90.0	90.0	80.0
**Maximum**	0	0	90.0	90.0	100.0	100.0	90.0	100.0	100.0	100.0
**Mean**	0	0	76.67	86.67	86.67	83.33	80.00	96.67	93.33	93.33
**SD**	0	0	23.09	5.77	15.28	20.82	10.00	5.77	5.77	11.55
**SE**	0	0	13.33	3.33	8.82	12.02	5.77	3.33	3.33	6.67
**Variance**	0	0	533.15	33.34	233.48	433.47	100.00	33.34	33.34	133.40
**F (DFn, DFd)**	F (9, 20) = 27.12									
**P value**	P<0.0001									

Table S7. Effect of salinity on deformity rate (%) of the larvae.

**Salinity**	**24**	**27**	**30**	**33**	**36**
**Minimum**	2.3	4.4	2.3	0.0	0.0
**Maximum**	6.5	7.0	4.7	2.3	2.4
**Mean**	4.4	5.3	3.8	0.8	0.8
**SD**	2.13	1.49	1.26	1.31	1.41
**SE**	1.23	0.86	0.73	0.76	0.81
**Variance**	4.52	2.23	1.59	1.72	1.99
**F (DFn, DFd)**	F (4, 10) = 5.450				
**P value**	P=0.0136				
